# Dual Mechanism for the Translation of Subgenomic mRNA from Sindbis Virus in Infected and Uninfected Cells

**DOI:** 10.1371/journal.pone.0004772

**Published:** 2009-03-10

**Authors:** Miguel Ángel Sanz, Alfredo Castelló, Iván Ventoso, Juan José Berlanga, Luis Carrasco

**Affiliations:** Centro de Biología Molecular “Severo Ochoa” (CSIC-UAM), Universidad Autónoma de Madrid, Cantoblanco, Madrid, Spain; Yonsei University, Republic of Korea

## Abstract

Infection of BHK cells by Sindbis virus (SV) gives rise to a profound inhibition of cellular protein synthesis, whereas translation of viral subgenomic mRNA that encodes viral structural proteins, continues for hours. To gain further knowledge on the mechanism by which this subgenomic mRNA is translated, the requirements for some initiation factors (eIFs) and for the presence of the initiator AUG were examined both in infected and in uninfected cells. To this end, BHK cells were transfected with different SV replicons or with *in vitro* made SV subgenomic mRNAs after inactivation of some eIFs. Specifically, eIF4G was cleaved by expression of the poliovirus 2A protease (2A^pro^) and the alpha subunit of eIF2 was inactivated by phosphorylation induced by arsenite treatment. Moreover, cellular location of these and other translation components was analyzed in BHK infected cells by confocal microscopy. Cleavage of eIF4G by poliovirus 2A^pro^ does not hamper translation of subgenomic mRNA in SV infected cells, but bisection of this factor blocks subgenomic mRNA translation in uninfected cells or in cell-free systems. SV infection induces phosphorylation of eIF2α, a process that is increased by arsenite treatment. Under these conditions, translation of subgenomic mRNA occurs to almost the same extent as controls in the infected cells but is drastically inhibited in uninfected cells. Notably, the correct initiation site on the subgenomic mRNA is still partially recognized when the initiation codon AUG is modified to other codons only in infected cells. Finally, immunolocalization of different eIFs reveals that eIF2 α and eIF4G are excluded from the foci, where viral RNA replication occurs, while eIF3, eEF2 and ribosomes concentrate in these regions. These findings support the notion that canonical initiation takes place when the subgenomic mRNA is translated out of the infection context, while initiation can occur without some eIFs and even at non-AUG codons in infected cells.

## Introduction

The genome of sindbis virus (SV), a member of the *Alphavirus* genus, contains a single-stranded RNA molecule of positive polarity [1]. After virus entry into susceptible cells and decapsidation the viral genome of 11.5 kb, acting as mRNA, directs the synthesis of early non-structural proteins (nsp1-4) involved in viral RNA replication and transcription. About 2–3 hours post-infection (hpi), synthesis of late SV proteins commences under the direction of 26S subgenomic (sg)-mRNA. This mRNA corresponds to the 3′ third of the genome and is transcribed from an internal promoter present on the minus strand RNA [2], [3]. After about 2 hpi, cellular translation is drastically inhibited, while viral sg-mRNA translation emerges and continues for hours. SV replicons encoding only the non-structural proteins are still capable of shutting off host translation [4]. Translation directed by sg-mRNA leads to synthesis of a polyprotein that is proteolytically cleaved, rendering the mature proteins C, E3, E2, 6K and E1. As occurs with the 49S RNA genome, the sg-mRNA is capped at its 5′ end and polyadenylated at the 3′ end. The cap structure is followed by 49-nt leader sequence then the AUG initiation codon. A translation enhancer element located in the first 275 nt of the C sequence confers high translatability on this sg-mRNA [5], [6]. In addition, this element is required for translation of sg-mRNA when eIF2α is phosphorylated [7]. Thus, significant phosphorylation of eIF2α is observed after togavirus infection, at times when structural proteins are synthesized [7], [8].

This modification of eIF2 is not responsible for the inhibition of host translation in SV-infected cells, since it occurs in cells where eIF2α is not phosphorylated [7], [9]. It is possible that the function of eIF2 is replaced by eIF2A in SV-infected cells [7]. As occurs with SV, a number of animal viruses are capable of translating their mRNAs in cells where eIF2 has become phosphorylated [10], [11]. In fact, some viral RNAs can direct the binding of Met-tRNA_i_ in an eIF2-independent manner [12], [13]. Although sg-mRNA contains a cap structure, cleavage of eIF4G by poliovirus (PV) 2A^pro^ or HIV-1 PR does not impair its translation [14]. These findings suggest that translation of SV sg-mRNA does not require the integrity of eIF4F complex and poly(A)-binding protein (PABP). A variety of animal viruses can translate some of their mRNAs in the absence of an integral eIF4F complex [10], [15]. One of the most studied examples is picornavirus mRNA, which directs protein synthesis after eIF4G cleavage by several viral proteases [16], [17]. In other examples, a virus-encoded protein interacts with eIF4G. Thus, influenza virus PB2 displaces eIF4E after its interaction with eIF4G [18], while adenovirus 100 K protein renders eIF4G unable to interact with Mnk1, leading to eIF4E dephosphorylation [19]. In addition, rotavirus NSP3 binds to both the 3′ end of viral mRNAs and eIF4GI, displacing its interaction with PABP and promoting circularization of rotavirus mRNAs [20], [21]. Although synthesis of SV late proteins does not need active eIF2, nor intact eIF4G or PABP, sg-mRNA in the cytoplasm of SV-infected cells will not necessarily be translated. This mRNA is not recognized by the protein-synthesizing machinery after its *in vitro* synthesis and electroporation in SV-infected cells. Moreover, when genuine sg-mRNAs synthesized in the infected cells are extracted and then electroporated back into the cells, they are excluded from translation in SV-infected cells but can direct protein synthesis in uninfected cells [22].

In the present work we show that SV sg-mRNA is translated according to the canonical model in uninfected cells or in cell-free systems, while this mRNA does not need certain initiation factors for translation when synthesized by the viral transcription machinery. In addition, a different initiation site is selected when mRNA is synthesized in the infected cells and when it is directly electroporated into uninfected cells or translated in cell-free systems. In both cases, the primary structure of SV sg-mRNA is the same, but the cellular context dictates the exact mode of initiation. We also show that, in infected cells, some translation factors and ribosomes migrate to transcription sites whereas others are excluded. These findings are consistent with the concept that translation is tightly coupled to transcription in virus infected cells [22].

## Results

### Intact eIF4G is necessary for translation of SV sg-mRNA in uninfected BHK cells, but is dispensable in SV-infected cells

**Figure 1:**
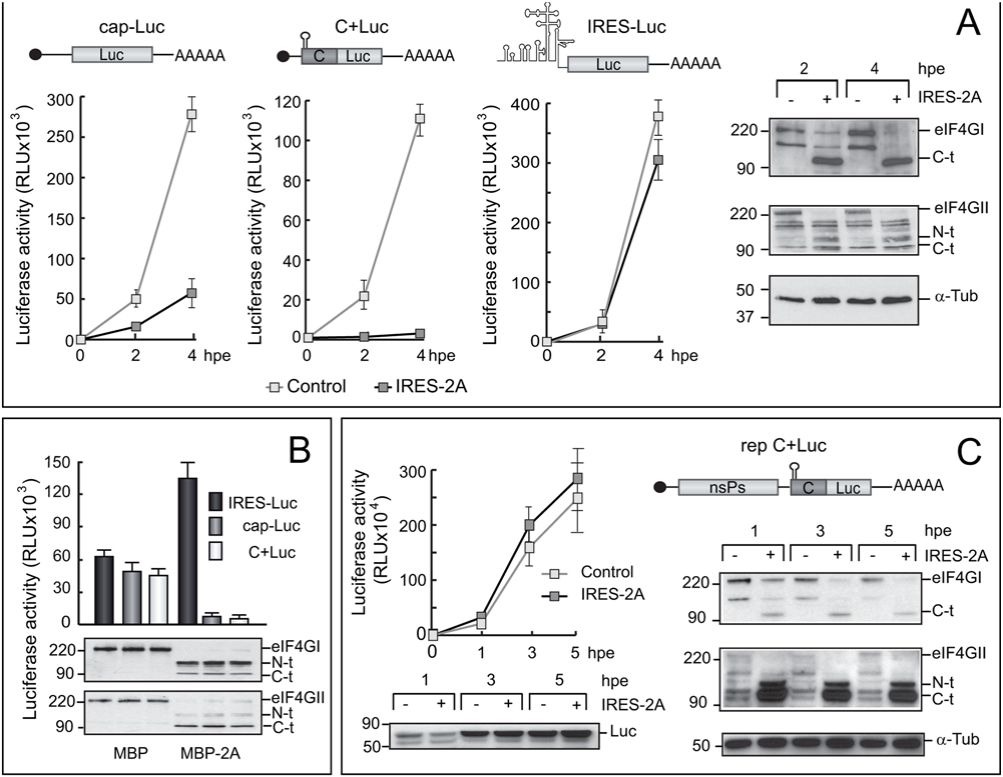
Requirement of eIF4G for translation of C+Luc sg-mRNA. Panel A. Protein synthesis and eIF4G cleavage in BHK cells. BHK cells were electroporated with 30 µg of IRES-2A mRNA or transcription buffer as a control. At 2 hpe, cells were again electroporated with 20 µg of C+Luc mRNA or cap-Luc-poly(A) or IRES-Luc-poly(A) as mRNAs control (mRNAs are schematized in the upper part of each figure). Values of luc activity obtained from the different mRNAs are represented. eIF4GI and eIF4GII integrity was analyzed by western blot analysis of extracts recovered at 2 and 4 hpe of IRES-2A. Tubulin was also analyzed to adjust the amount of each sample loaded onto the gel. Panel B. Translation directed by several mRNAs after the cleavage of eIF4G in HeLa S3 extracts. Translation was carried out in Hela S3 extracts pre-treated with purified MBP-2A or MBP proteins. Luc production was determined by measuring luc activity from each translation mixture and the integrity of eIF4GI and eIF4GII was analyzed by western blotting. Panel C. Effect of eIF4G cleavage on protein synthesis in BHK cells transfected with different SV replicons. BHK cells were electroporated with 20 µg of rep C+Luc mRNA (Schematized in the figure). At 2 hpe, cells were again electroporated with 30 µg of IRES-2A mRNA or transcription buffer. At 1, 3 and 5 hpe of IRES-2A mRNA, cell cultures were harvested and luc activity was measured or examined by western blotting with anti-luc antibodies (left panel). eIF4GI and eIF4GII integrity was tested by western-blot analysis. Tubulin was also analyzed to adjust the amount of each sample loaded onto the gel (right panel). α-Tub: α-Tubulin; luc: luciferase; C-t: C-terminal cleavage product of eIF4G; N-t: N-terminal cleavage product of eIF4G.

We previously reported that SV sg-mRNA can be translated when eIF4G is cleaved by viral proteases [14]. Initially two types of mRNAs were generated by *in vitro* transcription, that is, sg-C+Luc mRNA from pT7 C+Luc plasmid [22] and the replicon rep C+Luc [22]. After transfection, rep C+Luc gives rise to the sg-C+Luc mRNA using the SV transcription machinery. In the case of sg-C+Luc mRNA from pT7 C+Luc plasmid, after electroporation, sg-mRNA will be translated in uninfected cells and whereas in the other it will be translated in an environment that resembles the infected cells because there is viral replication and transcription. Translation of C+Luc mRNA renders a fusion protein that releases C protein and luciferase (luc) through proteolytic activity of C protein. To induce cleavage of translation initiation factor eIF4G, PV 2A^pro^ was expressed on electroporation of synthesized IRES-2A mRNA. This mRNA contains the EMCV IRES followed by the PV 2A^pro^ gene (IRES-2A) [23], [24]. BHK cells were then electroporated with IRES-2A mRNA or transcription buffer as control. At 2 hours post-electroporation (hpe), cells were once again electroporated with *in vitro* transcribed C+Luc mRNA using cap-Luc-poly(A) or IRES-Luc-poly(A) mRNAs as controls (Fig. 1A). eIF4GI and eIF4GII are both already proteolyzed by 2A^pro^ at 2 hpe (Fig 1A). Under these conditions cap-Luc-poly(A) mRNA translation was strongly inhibited at 4 hpe, suggesting that cap- and poly(A)-dependent translation was hampered in IRES-2A electroporated cells (Fig 1A). A similar situation occurs with C+Luc mRNA translation, which is also deeply inhibited after eIF4G cleavage (Fig 1A). These results support the idea that SV sg-mRNA is translated by a cap- and poly(A)-dependent mechanism in uninfected BHK cells. mRNAs that contain EMCV IRES are able to drive translation even when eIF4G is cleaved by 2A^pro^ [24]. Luc activity measured in cells electroporated with IRES-Luc increased consistently throughout the experimental period, irrespective of 2A^pro^-expression and eIF4G cleavage (Fig 1A). Similar findings were obtained in HeLa cells (data not shown). In addition, the effect of eIF4G cleavage on the translation of C+Luc mRNA was assayed in HeLa S3 cell extracts. To achieve eIF4G cleavage before mRNA addition, 1 µg of purified MBP-2A^pro^ was added to extracts for 30 min at 30°C, followed by translation of the different mRNAs. Luc activity was then estimated (Fig 1B, upper panel), along with hydrolysis of eIF4G (Fig 1B, lower panel). As with uninfected cells, cleavage of eIF4G strongly inhibits translation both of cap-Luc-poly(A) and C+Luc mRNAs whereas protein synthesis directed by IRES-Luc mRNA was stimulated.

We then determined the translational behaviour of C+Luc mRNA produced from the replicon rep C+Luc. In this case cells were first electroporated with rep C+Luc mRNA prepared *in vitro* and electroporated once again with IRES-2A mRNA two hours later. At 1, 3 or 5 hours after the second electroporation cultures were collected and luc production was determined by measuring luc activity (Fig 1C) and also by western blot analysis employing anti-luc antibodies (Fig 1C). The integrity of eIF4GI and eIF4GII was analyzed in parallel (Fig 1C). Translation of sg-C+Luc mRNA synthesized from the replicon rep C+Luc was not impaired when eIF4G was cleaved by 2A^pro^ expression (Fig 1C). These findings are in good agreement with the results obtained in SV-infected cells [14]. Taken together these data support the notion that SV sg-mRNA is translated differentially in infected and uninfected cells, as regards requirement for eIF4G integrity.

### Differential inhibition of SV sg-mRNA translation by arsenite in uninfected or SV-infected cells

**Figure 2:**
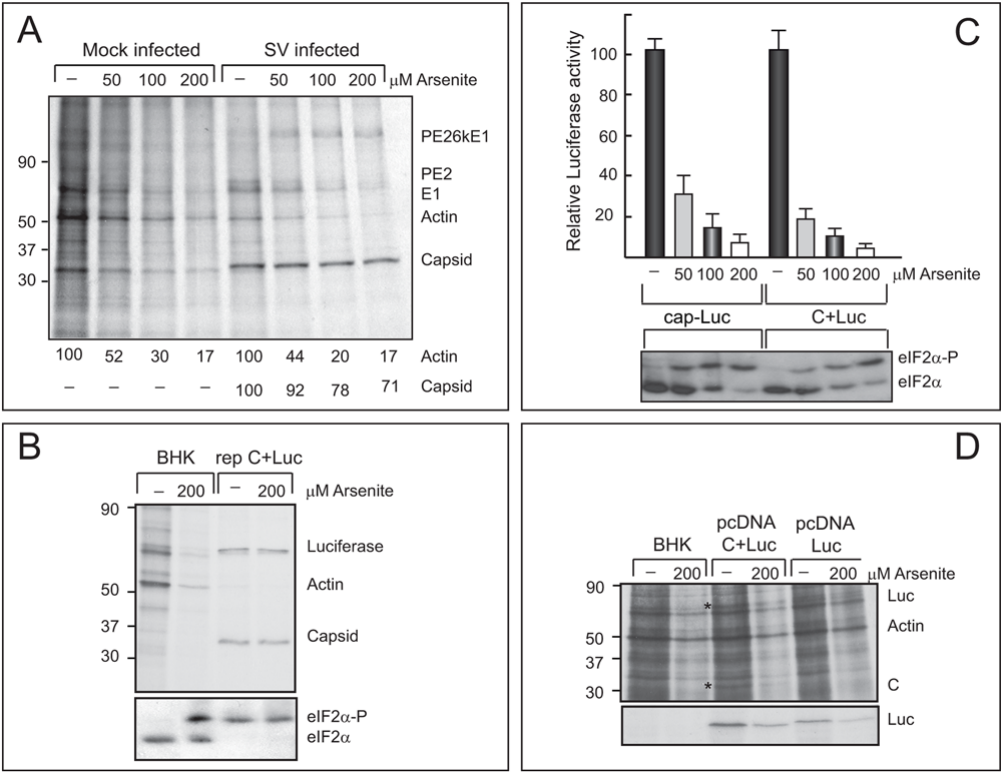
Effect of arsenite on translation of SV sg-mRNA. Panel A. Effect of arsenite on SV infection. Uninfected or SV-infected BHK cells (10 pfu/cell) were treated with different concentrations of sodium arsenite for 30 min at 4 hpi. Proteins were then labelled with [^35^S]Met-Cys in presence of the same concentrations of sodium arsenite for 30 min. Samples were collected in the appropriate sample buffer and processed by SDS-PAGE, fluorography and autoradiography. Relative densitometry values obtained from capsid or actin bands are indicated below each lane. Panel B. Translation in SV-replicating BHK cells treated with arsenite. BHK cells were electroplated with 20 µg of rep C+Luc mRNA or transcription buffer as control. At 4 hpe cultures were treated with sodium arsenite for 30 min and then half of the cultures were labelled with [^35^S]Met-Cys in presence or absence of arsenite for 30 min and the other half treated with arsenite only. Radioactive samples were examined by SDS-PAGE, fluorography and autoradiography (upper panel) and non-radioactive samples were used to analyze phosphorylation of eIF2α by isoelectric focusing (lower panel). Panel C. Translation of different mRNAs transfected in BHK cells treated with arsenite. BHK cells were electroporated with 20 µg of cap-Luc-poly(A) or C+Luc mRNA and at 30 mpe treated with different concentrations of arsenite for 1 hour. Half of the cultures were processed to measure luc activity (upper panel) and the other half to detect phosphorylation of eIF2 α by isoelectric focusing (lower panel). Panel D. Translation of sg-mRNA synthesized in the nucleus of BHK cells treated with arsenite. BHK cells were transfected with pcDNA-Luc or pcDNA C+Luc plasmids and, at 18 hpt, treated or not with arsenite for 30 min. Next, cultures were labelled with [^35^S]Met-Cys in presence or absence of arsenite for 30 min. One quarter of the samples were directly processed by SDS-PAGE, fluorography and autoradiography (upper panel). The remaining samples were first immunoprecipitated with anti-luc antibodies and then processed by SDS-PAGE, fluorography and autoradiography (lower panel).

Arsenite is widely used to induce phosphorylation of eIF2α, leading to the inhibition of translation [13], [25], [26]. Furthermore, culture cells infected by SV exhibit high levels of phosphorylated eIF2α at times when viral structural proteins are being synthesized [7], [9]. We therefore compared the eIF2α requirement for the translation of C+Luc mRNA both in uninfected and SV-replicating cells. First, we assayed the effect of arsenite on SV infection by analyzing protein synthesis in cultures treated with different concentrations of this compound (Fig 2A). Arsenite treatment blocked protein synthesis in control BHK cells in a dose-dependent manner. Thus, actin synthesis decreases by 48 and 83% on treatment with 50 and 200 µM arsenite respectively. However, in infected cells, synthesis of C protein is only reduced by 8 and 29%, respectively, after the same treatment. Moreover, as a consequence of arsenite activity at the endoplasmic reticulum, viral glycoprotein processing was affected, such that the precursor PE26KE1 was not cleaved to produce mature products PE2, 6K and E1.

The activity of arsenite on the translation of C+Luc mRNA was subsequently tested. To this end *in vitro* synthesized rep C+Luc or C+Luc mRNAs were electroporated into BHK cells. As with SV-infected cells, addition of arsenite had no effect on the translation of sg-mRNA derived from rep C+Luc (Fig 2B, upper panel). The phosphorylation state of eIF2α was analyzed in both control and rep C+Luc electroporated cells in the absence or presence of arsenite (Fig 2B, lower panel). This compound induced phosphorylation of eIF2α in control BHK cells. Moreover, in agreement with previous findings [7] almost total phosphorylation of eIF2α appeared in rep C+Luc transfected cells, both in absence or presence of arsenite.

The translation of C+Luc mRNA directly transfected into uninfected cells was quantified by measuring luc activity at 90 minutes post-electroporation (mpe). Interestingly, translation of C+Luc mRNA was strongly blocked by arsenite in uninfected cells (Fig 2C, upper panel) whereas, as shown above, this compound had little effect in cells electroporated with rep C+Luc (Fig 2B, upper panel). As a control, translation of a cap-Luc-poly(A) mRNA was strongly inhibited by arsenite as expected. The phosphorylation of eIF2α was analyzed in parallel (Fig 2C, lower panel). Arsenite treatment induced a dose dependent increase in the amount of phosphorylated eIF2α, while the unphosphorylated form diminished. Therefore, this phosphorylation induced by arsenite correlates with the inhibition of C+Luc and cap-Luc-poly(A) translation. Also, the action of dithiothreitol, another compound that induces eIF2α phosphorylation, was tested and the results were similar to those described for arsenite (results not shown).

In principle, the structure of C+Luc mRNA synthesized *in vitro* is similar to that produced by the SV transcriptional machinery, although the interactions of this mRNA with cellular proteins may vary according to whether it is directly electroporated into cells or synthesized inside BHK cells. To explore the effect of arsenite on the translational behaviour of C+Luc and cap Luc mRNAs transcribed in the nucleus, BHK cells were transfected with the pcDNA C+Luc or pcDNA Luc plasmids, which are substrates for RNA pol II that produce C+Luc and Luc mRNAs, respectively. At 18 hpe cultures were labelled with [^35^S]-Met/Cys in absence or presence of arsenite and luc was immunoprecipitated with anti-luc antibodies (Fig 2D). Arsenite treatment inhibits cellular translation (Fig 2D, upper panel) as well as luc synthesis (Fig 2D, lower panel). No differences exist between the inhibition by arsenite and luc synthesis from C+Luc or Luc mRNAs. Moreover, translation in HeLa S3 cell extracts was also assayed after the addition of poly I∶C to induce phosphorylation of eIF2α. As observed in uninfected cells, phosphorylation of eIF2α abrogated translation of both cap-Luc-poly(A) and C+Luc mRNAs (data not shown). In conclusion, eIF2α is not necessary for the translation of mRNA C+Luc in SV-replicating cells, but this initiation factor is required for the translation of sg-mRNA in contexts other than infection.

### Translation of sg-mRNA variants lacking the AUG initiation codon

**Figure 3:**
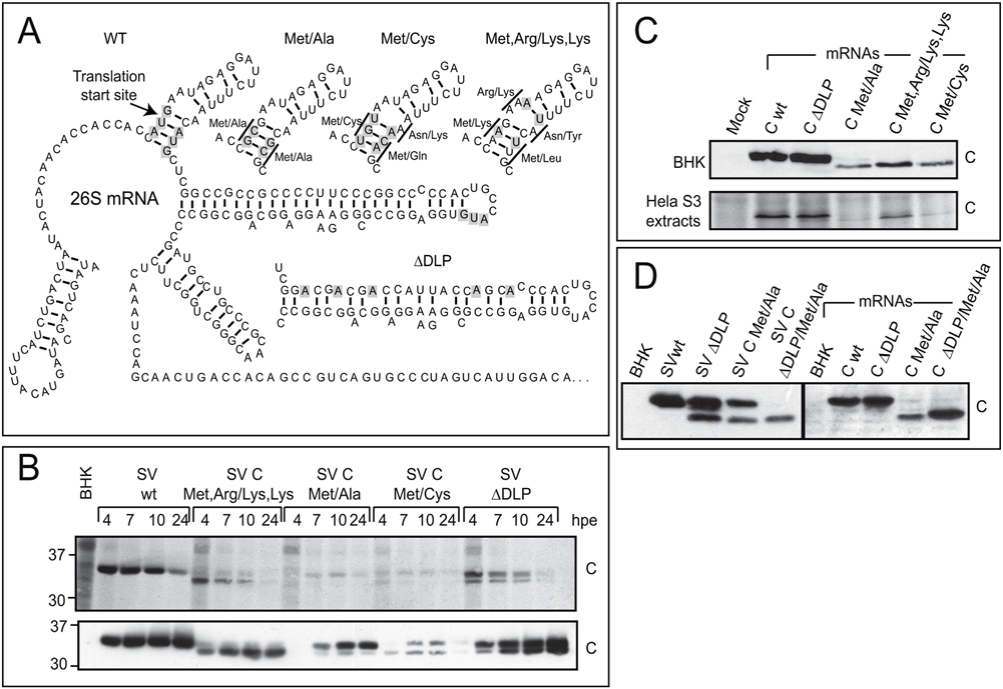
Translation of SV sg-mRNA containing AUG_i_ replaced by other codons. Panel A. Schematic representation of the first 228 nt from the 5′end of SV sg-mRNA that include the leader sequence and the translation enhancing motif. The mutations introduced in the different constructs are indicated. Panel B. Synthesis of C protein from different SV variants with modified start codons of sg-mRNA. BHK cells were electroporated with the different *in vitro* transcribed mRNAs and, at the times indicated, cultures were labelled with [^35^S]Met-Cys for 30 min. Samples were processed by SDS-PAGE, fluorography and autoradiography (upper panel) and also by western blot analysis to detect C products with anti-C antibodies (lower panel). Panel C. Synthesis of C protein from sg-mRNAs with altered AUG_i_ electroporated into BHK cells or translated in HeLa cell extracts. BHK cells were electroporated with the different *in vitro* prepared sg-mRNAs and, at 3 hpe, C production was analyzed by western blotting (upper panel). Translation was carried out in HeLa S3 extracts programmed with the different mRNAs for 1 h at 30°C in presence of 0.7 µC/µl [^35^S]Met-Cys. The synthesized proteins were analyzed by autoradiography of SDS-polyacrylamide gels (lower panel). Panel D. Synthesis of C protein from sg-mRNAs that have disrupted the DLP structure. BHK cells were electroporated with different SV genomes or with sg-mRNAs synthesized by *in vitro* transcription. Cultures were collected at 4 hpe for replicons and at 3 hpe for *in vitro* transcribed sg-mRNAs. C products were analyzed by western blotting with anti-C antibodies. Samples electroporated with sg-mRNAs (Right panel) were exposed ten times more than samples electroporated with replicons (Left panel).

Since translation of sg-mRNA does not require eIF2 in SV-infected cells, we speculated that a different initiation codon to the canonical model may be used for this mRNA. Perhaps, during infection, when eIF2α is phosphorylated the initiation of translation of sg-mRNA could operate at non-AUG codons. To investigate this possibility, a number of constructs were made containing AUG_i_ and the next AUG present in the region coding for C protein changed to other codons. To maintain the predicted base pairing in this region, several modifications were introduced as depicted in figure 3A. In addition, we tested a variant that contains an altered hairpin structure (SV ΔDLP) [7]. The different viral genomic RNAs bearing altered initiation codons and the variant that contains a modified hairpin structure were electroporated into BHK cells and protein synthesis was estimated at different times by radioactive labelling (Fig 3B, upper panel) and also by western blot analysis using anti-C antibodies (Fig 3B, lower panel). As expected, wt SV RNA was the most efficient and synthesized C as a unique product. SV ΔDLP rendered three products that in total represent 28% of C product from wt SV, as estimated by densitometric analysis of the labelled sample at 10 hpe. The two products with higher molecular weight migrate very closely to each other, but they can be separated by using longer electrophoresis times (data not shown). Most probably, these C-related products are the result of a loss of fidelity in AUGi selection during the initiation of translation. Their sizes are consistent with alternative initiation at the first three AUGs of C sequence. The SV-C-Met,Arg/Lys,Lys variant rendered two products, the one with the lower mobility corresponds in size to wt C and represents 7% of protein C synthesized at 10 hpe. Gel mobility of this product suggests that it is synthesized by initiation at the non-canonical AAG codon (see scheme in Fig 3A). The most abundant C product synthesized by SV-C-Met,Arg/Lys,Lys (19% of wt C) is consistent with a translation initiation from the first AUG of its sequence, that is the third AUG in the wt C (see scheme in Fig 3A). SV-C-Met/Ala and SV-C-Met/Cys synthesized the lower amounts of C products at 10 hpe, 9 and 5% of wt C respectively. However, they synthesized almost exclusively products with similar mobility to wt C protein. These C products are presumably derived from initiation at non-canonical GCG or UGU codons, respectively. Analyses and quantification by western blot analysis yielded similar results to those obtained by radioactive labelling (Fig 3B, lower panel). However, one significant difference was that the products synthesized by translation initiation at the non-canonical codons AAG from SV-C-Met,Arg/Lys,Lys or UGU from SV-C-Met/Cys do not accumulate in cells. As wt C protein does not contain any cysteine, this variant will produce a radioactive C product when labelled with radioactive cysteine if the UGU codon is used. BHK cells electroporated with wt SV and SV-C-Met/Cys RNAs were labelled at 5 hpe with [^35^S]-Cys (Fig S1). wt SV efficiently incorporated radioactive cysteine into its glycoproteins but not into C protein. Notably, although inefficiently incorporated radioactive cysteine into SV-C-Met/Cys, it could be detected both in glycoproteins and in C protein. The mobility of the labelled C protein suggests that translation initiates at the UGU codon.

We subsequently examined C synthesis using sg-mRNAs prepared *in vitro* in both uninfected cells and Hela S3 extracts. In the first case the different sg-mRNAs were electroporated into BHK cells and, at three hpe, C production was determined by western blot analysis (Fig 3C, upper panel). In the second case translation was examined by radioactive labelling of Hela S3 extracts programmed with different mRNAs (Fig 3C, lower panel). The results from both experiments coincide. Only one product is synthesized from each mRNA analyzed and these products are always generated from translation initiation at the first AUG present at their respective sequences. Once again, as occurred after eIF4G cleavage or arsenite treatment, a different translational behaviour of sg-mRNA is observed according to whether it is transcribed by the viral machinery or directly electroporated into uninfected cells or translated in HeLa cell extracts. In conclusion, canonical initiation takes place when the sg-mRNA is translated out of the infection context, while perhaps initiation may occur even at non-AUG codons during infection. Modifications of AUG_i_ to other codons in hepatitis C virus mRNA has little effect on its translability [27]. In this regard, SV sg-mRNA shares some similarities with the behaviour observed in hepatitis C virus mRNA.

Although Met/Ala mutant is a weak initiator at GCG, it is notable that this variant is still capable of significantly recognizing the GCG codon. We reasoned that the hairpin present in the C sequence probably pointed to the site where initiation starts, even if the AUG codon has been replaced by GCG. To analyze this possibility a new construct was generated that combines ΔDLP and Met/Ala mutations. BHK cells were electroporated with the different replicons prepared *in vitro* and C production was analyzed by western bloting (Fig 3D, left panel). Significantly, synthesis of C protein with the new variant SV-C-ΔDLP/Met/Ala differs from SV-C-Met/Ala because only the fastest migrating C product is synthesized. Therefore, disruption of DLP structure abrogates translation initiation at the non-canonical GCG codon. Thus, the DLP structure could signal the translation start codon in the infection context. In agreement with the above experiments one product could be detected (Fig 3D, right panel) when translation of this new sg-mRNA was tested in uninfected cells only. Notably, truncated C production was higher from C –ΔDLP/Met/Ala mRNA than from C-Met/Ala mRNA. Perhaps, because the first AUG from C-Met/Ala mRNA is in the hairpin sequence that contains a very stable secondary structure (see Fig 3A) in a canonical initiation, this hairpin may hamper the scanning process to select the AUGi, whereas the non-structured ΔDLP sequence would make the AUGi more accessible.

### Location of several eIFs and ribosomes in SV-infected cells

**Figure 4:**
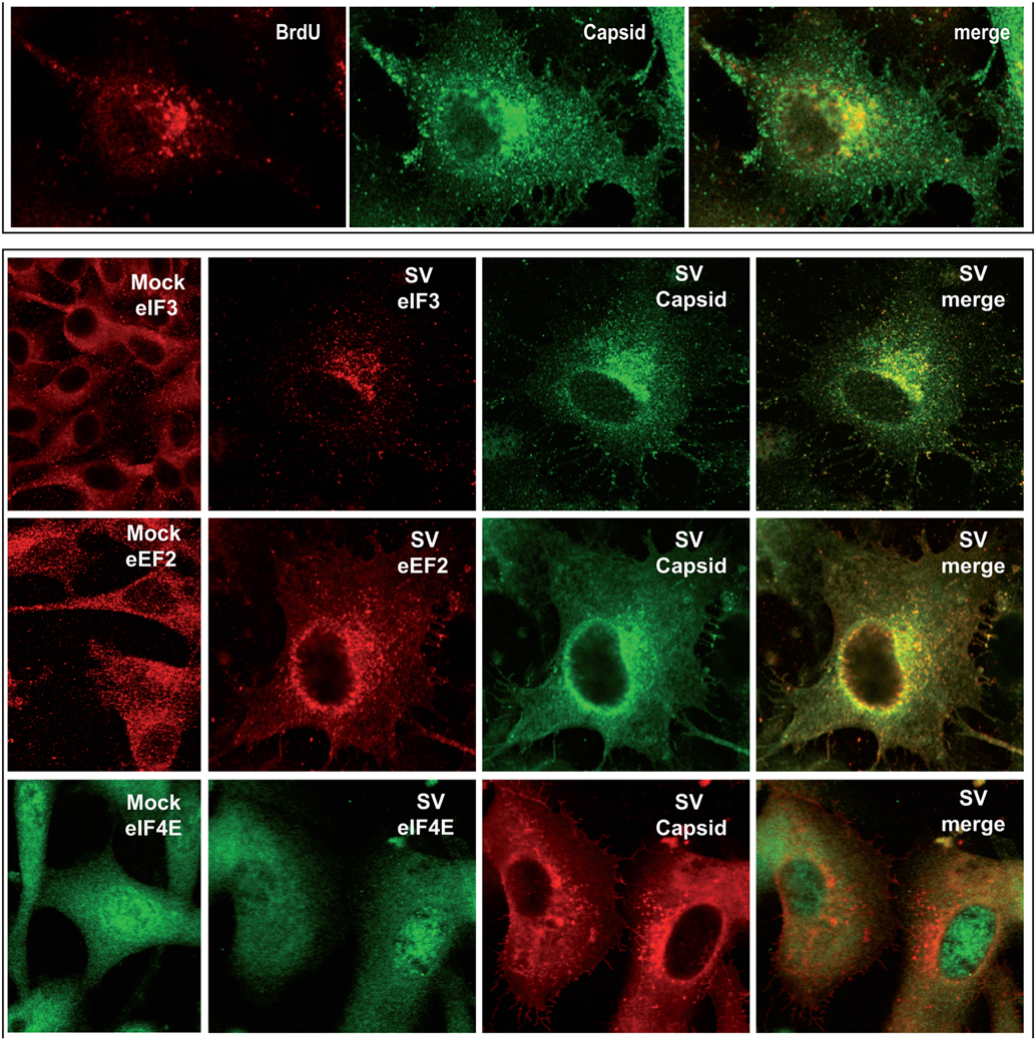
Subcellular localization of SV replicative complexes and different viral and cellular proteins. Panel A. Co-localization of viral transcription complexes and nucleocapsid aggregation sites in SV-infected cells. BHK cells were infected with SV (100 pfu/cell) and, at 7 hpi, the medium was supplemented with dactinomycin (2.5 µg/ml) for one hour. Cells were then transfected with a mixture of bromouridine (10 mM) and Lipofectamine 2000 reagent for 30 min. After this time, the transfection medium was replaced by 10% FCS supplemented with dactinomycin for 30 min. Immunofluorescence analysis was carried out as described in Materials and Methods. Panel B. Co-localization analyses of capsid protein and translation factors in SV-infected cells. BHK cells were infected with SV (100 pfu/cell) and, at 8 hpi, cells were processed for immunofluorescence.

**Figure 5:**
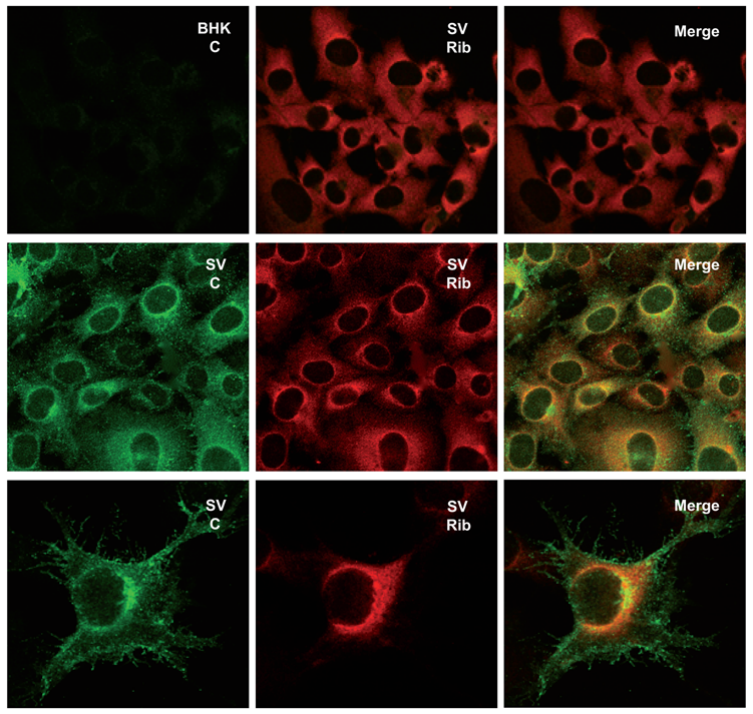
Co-localization analyses of SV capsid protein and ribosomes. BHK cells were infected with SV (100 pfu/cell) and processed for immunofluorescence at 8 hpi.

We recently provided evidence that transcription and translation are coupled in SV-infected cells [22]. In such a case, we would predict that viral translation takes place in cytoplasmic regions close to transcription and replication factories. Indeed, electron microscopy of SV-infected cells shows that nucleocapsids are assembled around membranous structures localized at discrete sites in the cytoplasm (Fig S2). Some of these membranous structures resemble replication factories (Fig S3). Our first goal was therefore to determine whether viral nucleocapsids co-localize with active transcription sites. To this end, SV-infected cells were labeled with bromouridine (BrU) in presence of actinomycin D at 6 hpi. Fixed cells were then incubated with specific antibodies against C protein and BrU and analyzed by immunofluorescence (Fig 4, upper panel). Transcription sites detected by anti-BrU antibodies did indeed co-localize with C protein. The next step was to examine co-localization of different translation factors with C protein (Fig 4, lower panel). The subcellular distribution of p-110 subunit of eIF3 in SV-infected cells differs from that observed in uninfected BHK cells. In uninfected cells eIF3 is uniformly distributed throughout the cytoplasm, whereas this factor is concentrated in the region of the nucleus in SV-infected cells. Moreover, eIF3 co-localizes with SV C protein. Translation elongation factor eEF2 has a localization and behaviour similar to eIF3, whereas eIF4E does not modify its distribution. To determine whether ribosomes are redistributed during infection, a monoclonal antibody against the carboxy terminal end of P ribosomal protein was employed. Figure 5 shows that ribosomes appear concentrated near the nucleus in SV-infected cells but are spread throughout the entire cytoplasm in control BHK cells. In SV-infected cells, most of the cytoplasm is devoid of ribosomes, which are concentrated close to the nucleus, near and overlapping the C protein signal (see in more detail lower panels in Fig 5). These findings suggest that components of the protein synthesizing machinery are redistributed after SV infection, localizing to a perinuclear region enriched in C protein, where viral transcription is taking place.

**Figure 6:**
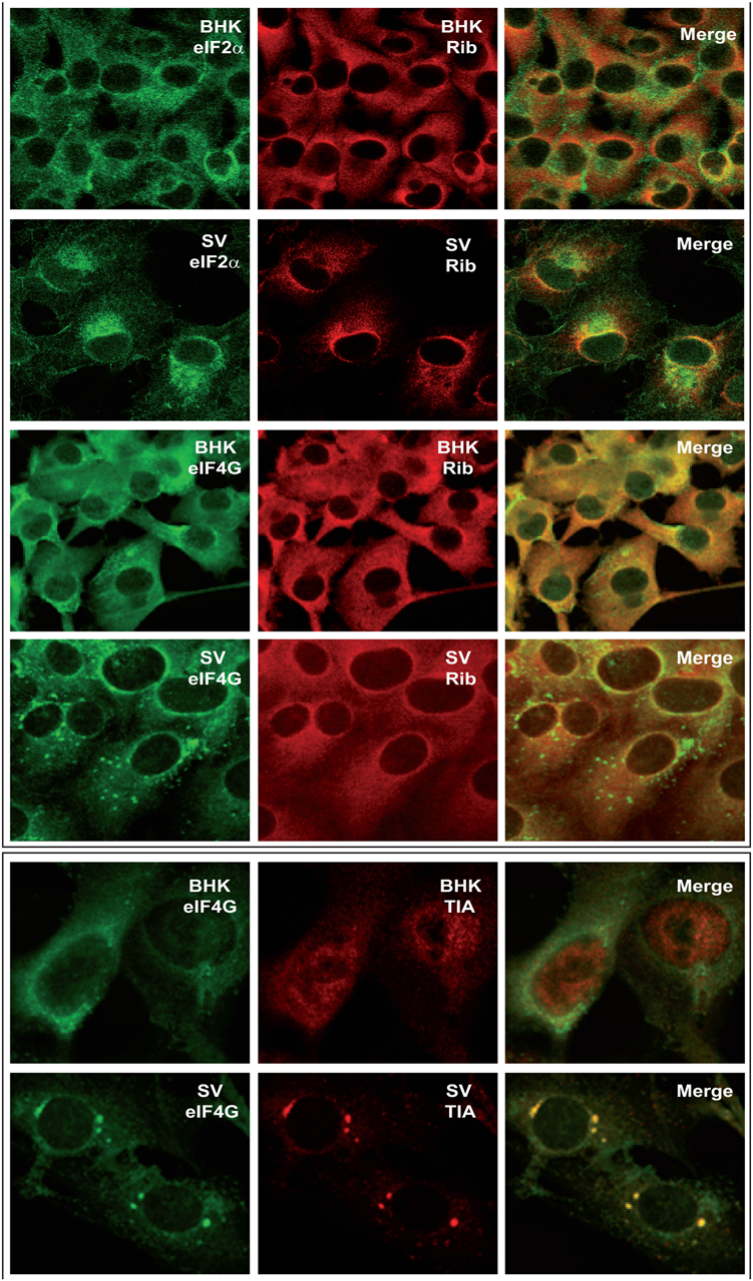
Localization analyses of eIF2α and eIF4G in SV-infected cells. BHK cells were infected with SV (100 pfu/cell) and were processed for immunofluorescence at 8 hpi.

As shown in this work and in previous articles, synthesis of SV late proteins in infected cells can take place without operative eIF4G and eIF2α [7], [14]. For this reason, it was of interest to analyze the distribution of these initiation factors after SV infection. Both eIF2α and eIF4GI modified their location in SV-infected cells, as compared to the uninfected counterparts. Thus, eIF2α concentrates in a region near the nucleus devoid of ribosomes, presumably the centrosomal region (Fig 6, upper panel). On the other hand, most of eIF4GI is found in cytoplasmic granules (Fig 6, upper panel), which are most probably stress granules since both eIF4GI and TIA markers co-localize in SV-infected cells (Fig 6, lower panel). The presence of eIF2α and eIF4GI in places other than those enriched in ribosomes and other translation factors is consistent with the idea that these two factors do not participate in the initiation of translation of SV sg-mRNA.

## Discussion

Viruses have evolved special mRNA structures that confer high translatability under conditions where cellular protein synthesis has been abated. The most studied of these structures are IRESs, which direct the internal initiation of translation instead of the typical cap recognition step [16], [28], [29]. Viral mRNAs that contain an IRES element exhibit special requirements as regards the integrity of initiation factors. In particular, eIF4G cleaved by some picornavirus or retrovirus proteases can still participate in the initiation of translation on mRNAs bearing an IRES element [30], [31]. Interestingly, SV sg-mRNA, which is devoid of an IRES and contains a cap structure at its 5′ end, is still translated when eIF4G is cleaved by poliovirus 2A^pro^ or HIV PR [14]. Most notably, we now present evidence that the SV sg-mRNA requires eIF4G to be intact when it is translated in uninfected cells or in cell-free systems. These findings support the idea that this sg-mRNA is translated following the canonical mechanism in uninfected cells, such that there is a cap recognition step. Cleavage of eIF4G abrogates the cap recognition necessary for translation of SV sg-mRNA in uninfected cells. Surprisingly, the eIF4F complex and therefore cap recognition do not seem to be operative in SV-infected cells during late viral protein synthesis. A similar picture also emerges for the participation of eIF2 during sg-mRNA translation. This factor is not required for the translation of SV sg-mRNA because it is highly phosphorylated in the infected cells. However in uninfected cells or in cell-free systems, phosphorylation of eIF2 induced by arsenite or poly I∶C treatment strongly blocks protein synthesis directed by SV sg-mRNA. The new concept that arises from these findings is that the structure of a given mRNA does not suffice to dictate its translation mechanism. Thus, a viral mRNA, such as SV sg-mRNA, follows the canonical mechanism for the initiation of translation in uninfected cells or in cell-free systems, whereas the same mRNA exhibits different eIF requirements in virus infected cells. Therefore, some viral mRNAs may exhibit a dual mechanism for their translation, i.e. the requirements for eIFs observed in cell-free systems may differ from those found in the infected cells. If so, a reappraisal of previous experiments studying the mechanisms of viral mRNA translation in transfected cells or in cell-free systems should be undertaken.

More surprises about the mechanism of initiation on the SV sg-mRNA appeared when AUG_i_ was modified. Changes of AUG to other codons still allowed recognition of the original start site on the sg-mRNA, although the efficacy of translation diminished. The Met to Ala modification (AUG/GCG) was more efficiently recognized than the other modifications tested, but the important observation is that SV sg-mRNA still serves to initiate protein synthesis, even without an AUG_i_. These findings are in good agreement with the observation that hepatitis C virus mRNA is translated even when the initiator AUG codon is replaced by other codons [27]. Notably, Met/Ala SV sg-mRNA is unable to start translation at that site in uninfected cells or in cell-free systems and translation start at the first AUG codon. These results suggest that a different start codon is selected according to whether the mRNA has been transcribed in SV-replicating cells or in uninfected cells or in cell-free systems. Since the structure of the SV sg-mRNA is similar in both cases, i.e. infected or uninfected cells, we can conclude that the exact mechanism of translation of this mRNA depends on the context in which protein synthesis is examined. Therefore, the plasticity of the translation machinery is adapted to a given mRNA according to the environment.

The hairpin loop present in the coding region of the SV sg-mRNA sequence is a translational enhancer [5], [6], although its exact function during translation remains puzzling. In addition, this enhancing structure is crucial to signal the translation start codon, since its base pairing disruption gives rise to low accuracy for AUG_i_ selection [5], [7]. Similar elements have also been described for dengue and West Nile viruses [32], [33]. In fact, it has been proposed that the hairpin element present in the capsid coding region of the dengue virus directs AUG selection [32]. Consistent with these findings, disruption of the hairpin structure in the Met-Ala variant abolishes the recognition of GCG and protein synthesis in this variant starts at the first AUG present in the sg-mRNA. Although inefficient, this mechanism used by SV sg-mRNA for initiation at a non-AUG codon is intriguing. We are tempted to speculate that perhaps this hairpin structure acts in a way akin to other structures found in IRES sequences; particularly the one studied with the dicistrovirus IRES [34], [35]. In this case, a hairpin mimics a deacylated tRNA able to interact with the empty P site of the ribosome. The potential advantage for efficient translation conferred by the hairpin structure on sg-mRNA compared to mRNAs may provide new clues that help understand the molecular mechanism of the shut-off of host translation. Thus, certain eIFs might not be needed for sg-mRNA translation because of a viral protein. Both the sg-mRNA structure and viral proteins would determine the mechanism by which this viral mRNA is translated.

Another point of interest that should be taken into account to interpret our present results is the coupling between transcription and translation of viral mRNAs [22], [36], [37], [38]. Thus, in SV-infected cells the only mRNAs that are translated in the late phase are those synthesized by the viral transcription machinery, while electroporated viral mRNAs with the same structure are ignored by the translational apparatus [22]. Several years ago it was proposed that cytopathic vacuoles constitute the sites where not only viral RNA synthesis but also translation takes place in SV-infected cells [39]. Certainly, we have now observed by electron microscopy that viral nucleocapsids are assembled at membranous structures in close connection with replication factories. Some of these membranous structures have invaginations that form spherules previously described as cytopathic vacuoles I. These vacuoles have numerous capsids attached and are located at the same sites as replicative complexes (Fig S3). Apart from these places very low C signal was evidenced in the cytoplasm by immuno-gold analyses (Fig S2). The co-localization of replication complexes and C protein was also determined by confocal microscopy (Fig 4A). In addition, our present findings reveal that some translation factors as eIF3 or eEF2 and ribosomes co-localize with the nucleocapsid aggregation sites (Fig 4B). This phenomenon may be due to the fact that protein synthesis takes place at discrete foci in SV-infected cells, where ribosomes are recruited and coincide with the sites in which viral genome replication is taking place. Consistent with our results, proteomic studies of replicative complexes evidence the presence of ribosomal proteins [40]. By contrast, eIF4G and eIF2 are excluded from SV replication sites, such that eIF4G localizes to stress granules as occurred in SFV-infected cells [8], while eIF2 presumably appears in the centrosomal region. The findings that viral transcription and translation are coupled and take place at discrete cytoplasmic regions, agrees well with the recent findings on vaccinia virus infected cells [41], [42]. However, it should be stressed that the mechanism of viral translation may differ according to the animal virus considered. For instance eIF2 and eIF4G are excluded in SV-infected cells but could be necessary for translation of vaccinia virus mRNAs. In summary, it seems that translation of SV genomic mRNA gives rise to the formation of replication factories in modified membranous vesicles early during infection. These vesicles are concentrated in a perinuclear region as infection progresses. Translation components such as ribosomes and some factors accumulate close to these vesicles where they can participate in viral protein synthesis. Thus, viral transcription produces genomic RNA prone to encapsidation and sg-mRNA translation in a limited cellular space, favouring the interactions between viral RNAs and different cellular and viral proteins.

## Materials and Methods

### Cell line and viruses

Baby hamster kidney (BHK-21) cells and SV were used to perform the experiments. SV virus stock was prepared from a pT7 SVwt infective cDNA clone (where wt is wild type) [43]. Viral infection of BHK cells was carried out in Dulbecco's modified Eagle medium (DMEM) without serum for 40 min to permit virus attachment. This medium was then removed and infection continued in DMEM with 10% fetal calf serum.

### Plasmids and recombinant DNA procedures

pT7 SVwt was used as the parental plasmid for all of the constructs. pT7 rep C+Luc and pT7 C+luc [22], pKs-Luc and pTM1-2A [23], pcDNA-Luc [44] and pTM1-Luc [45] have been described previously. pcDNA C+Luc was generated by cloning the sg-C+Luc sequence from pT7 rep C+Luc in pcDNA. To this end a double PCR product digested with Mlu I and Xho I was inserted into the same sites of pcDNA. The double PCR product was prepared as follows: for the first PCR primers 5′Mlu pcDNA and 3′joint pcDNA-L26S (the sequences are shown below) were used with pcDNA as DNA template, and for the other PCR, primers 5′joint pcDNA-L26S and 3′Xho SV were used with pT7 rep C+Luc as DNA template; a mixture of these products and 5′Mlu I pcDNA and 3′Xho SV as primers were then used for the second PCR. Mutants in the AUGi pT7 SV-C-Met, Arg/Lys,Lys; pT7 SV-C-Met/Ala and pT7 SV-C-Met/Cys were prepared by inserting the corresponding double PCR product digested with Hpa I and Aat II enzymes into the same sites of pT7 SVwt. pT7 SVwt was always used as DNA template and the oligonucleotides employed are shown below. The first PCR products were obtained using 5′Hpa I SV as 5′ oligonucleotide and one of the different 3′mut oligonucleotides as 3′ oligonucleotide; the other PCR product was prepared using one of the different 5′mut oligonucleotides as 5′ oligonucleotide and 3′Aat II SV as 3′ oligonucleotide. The double mutant pT7 SV ΔDLP/Met/Ala was prepared in the same way, but using 5′Hpa I SV and 3′mut Met/Ala oligonucleotides with pT7 SV ΔDLP as DNA template and 5′mut Met/Ala and 3′Aat II SV with pT7 SV-C-Met/Ala as DNA templates for the first PCRs. The constructs pT7 C-wt, pT7 C-ΔDLP, pT7 C-Met/Ala and pT7 C-ΔDLP/Met/Ala were designed to produce the corresponding sg-mRNAs by *in vitro* transcription. To this end we used the oligonucleotides 5′SacI-T7prom and 3′Aat II SV and the different pT7 SV constructs as DNA template. These PCR products were subsequently digested with Sac I and Aat II enzymes and inserted into the same sites of pT7 SV rep C [46].

### Oligonucleotides

5′Mlu I pcDNA: ccgatatacgcgttac; 3′joint pcDNA-L26S: gaaagttactatgctgactagttagccag agagctctg; 5′joint pcDNA-L26S: cagagctctctggctaactagtcagcatagtacatttc; 3′Xho SV: atta attcccctcgaggaattccc; 5′Hpa I SV: ggccgggcccgttaaccggtctgatgatc; 3′Aat II SV: gttcttgac gtcgaacaatct;5′mut Met/Ala: caccaccgcgaatagaggattctttaacgcgctcggcc; 3′mut Met/Ala: g gccgagcgcgttaaagaatcctctattcgcggtggtg; 5′mut Met,Arg/Lys,Lys: caccaagaaaggattcttttact tgctcggccgc; 3′mut Met,Arg/Lys,Lys: ggcggagcaagtaaaagaatcctcttttcttggtggtg; 5′mut Met/Cys: caccaccacctgtaatagaggattctttaaacagctcggccgcc; 3′mut Met/Cys: ggcggccgagct gtttaaagaatcctctattacaggtggtggtg; 5′SacI-T7prom: gcgcgcgagctctaatacgactcactatagatagtc agcatagt.

### *In vitro* transcription and transfection

Plasmids were used as templates for *in vitro* RNA transcription with T7 RNA polymerase (Promega). The transcription mixture always contained an m^7^G(5′)ppp(5′)G cap analog except when mRNAs containing EMCV IRES were prepared. When pKS-Luc, pTM1-Luc or pTM1-2A plasmids were used as templates, *in vitro* polyadenylation was performed with poly(A) polymerase (Invitrogen). For transfection, subconfluent BHK cells were harvested, washed with ice-cold phosphate-buffered saline, and resuspended at a density of approximately 2.5×10^6^ cells/ml in the same buffer. Subsequently, 20 µg of *in vitro*-transcribed RNA were added to 0.4 ml cell suspension and the mixture was transferred to a 2-mm cuvette. Electroporation was carried out at room temperature by generating two consecutive 1.5-kV, 25-mF pulses with a Genepulser apparatus (Bio-Rad), as previously described [47]. Plasmids were transfected using Lipofectamine 2000 reagent (Promega) as recommended by the supplier.

### *In vitro* translation in HeLa S3 cell extracts

Translations were carried out in HeLa S3 cell extracts treated with micrococcal nuclease (a gift from E. Wimmer, Department of Molecular Genetics and Microbiology, Stony Brook University, NY, USA) and programmed with mRNAs synthesized by *in vitro* transcription. The preparation of extracts was essentially as described by Molla et al. [48]. Reactions were incubated at 30°C for 30 min in presence of 1 µg of MBP-2A [49] for each 10 µl HeLa S3 cell extracts to induce cleavage of eIF4G or in presence of 1 µg of MBP as control. To induce phosphorylation of eIF2α, extracts were treated with 0.5 µg/ml poly I∶C also for 30 min. Subsequently, 100 ng of different transcript RNAs were added and reactions left to run for 1 hour at 30°C. Protein production was estimated by measuring luc activity. In the case of radioactive labelling, 0.7 µCi/µl of [^35^S]Met-Cys was added to the reaction mix and the synthesized proteins were analyzed by autoradiography of SDS-polyacrylamide gels.

### Analysis of protein synthesis by radioactive labelling

BHK cells were seeded into 24-well plates at a concentration of about 10^5^ cells/well. At the times indicated for each experiment, the media were removed and proteins were labelled for 30 min with 0.2 ml DMEM without methionine-cysteine supplemented with 2 µl EasyTag™ EXPRESS ^35^S Protein Labeling mix, [^35^S]Met-Cys (14 mCi/ml; Perkin Elmer) per well or with 0.2 ml DMEM without cysteine supplemented with 4 µl [^35^S]Cys (1 mCi/ml; Perkin Elmer) for 60 min. The cells were then collected in the appropriate gel loading buffer and analyzed by autoradiography of SDS-polyacrylamide gels.

### Measurement of luciferase activity

Cells were lysed in a buffer containing 0.5% Triton X-100, 25 mM glycylglycine (pH 7.8), and 1 mM dithiothreitol. Luciferase activity was determined using a Monolith 2010 luminometer (Analytical Luminescence Laboratory), using the Luciferase Assay System (Promega). Luc activity results are means±s.d. of three representative experiments performed in triplicate.

### IEF and western blot analysis

Isoelectric focusing was carried out as described [7], [50]. Rabbit anti-eIF2α antibody (Santa Cruz) was used to detect eIF2α. The other antibodies used in western blot experiments were rabbit antisera raised against firefly luciferase (Promega), rabbit anti-eIF4GI [51], rabbit antisera raised against the N-terminal and C-terminal region of eIF4GII (a gift from N. Sonenberg, McGill University, Montreal, Canada), and monoclonal anti-α-tubulin (Sigma). Moreover, anti-C antibody was obtained by immunization of a rabbit with purified nucleocapsids from SV infected BHK cells.

### Cell processing for electron microscopy (EM)

At 8 hpi, cells were fixed with 2% glutaraldehyde in 0.2 M HEPES buffer (pH 7.4) for 1 h at room temperature and immediately scraped off the plate. For conventional EM, cells were washed twice and resuspended in 0.2 M HEPES buffer (pH 7.4). After fixing, dehydrating, and infiltrating the cells with Epon, thin sections were obtained and stained with uranyl acetate and lead citrate. For immunoelectron microscopy, cells were processed by freeze substitution. Immunogold localization of SV capsid protein was done by placing the ultrathin sections on drops of different solutions. After a 30-min incubation with TBG (TBS (Tris-ClH 30 mM, ClNa 150 mM, pH 8.2) plus 0.1% BSA and 1% cold water fish skin gelatine), sections were floated for 1 h on a drop of anti-C antibodies diluted in TBG. Next grids were washed in TBS plus 0.1% BSA (3×5 min) and then exposed to 10 nm colloidal gold conjugated goat anti-rabbit IgG diluted in TBG for 1 hour. Grids were then washed consecutively with TBG, TBS, and distilled water (5 min each) before staining with a saturated solution of uranyl acetate followed by lead citrate.

### Immunofluorescence microscopy

BHK cells were seeded on coverslips and infected with SV (100 pfu/cell). At 8 hpi cells were fixed in 4% PFA for 15 min, washed twice with PBS, and then permeabilized for 10 min with 0.2% Triton X-100 in PBS. All antibody incubations were carried out for 1 h in PBS containing 0.1% FCS and 0.1% Triton X-100. Coverslips were washed three times with PBS between primary and secondary antibody incubations, mounted in ProLong Gold anti-fade reagent (Invitrogen) and finally examined with a Radiance 2000 (Bio-Rad/Zeiss) confocal laser scanning microscope. Primary antibodies used were mouse monoclonal anti-bromodeoxyuridine BrdU (Affinity BioReagents), rabbit polyclonal anti-C, mouse monoclonal against the carboxy terminal end of P ribosomal protein [52] (a gift from J.P. García Ballesta, Centro de Biología Molecular Severo Ochoa, Madrid, España), goat polyclonal anti-eIF3 p110 (C-20) (Santa Cruz Biotechnology, Inc.), goat polyclonal anti-EF-2 (P-19) (Santa Cruz Biotechnology, Inc.), mouse monoclonal anti-eIF4E (P-2): sc-9976 (Santa Cruz Biotechnology, Inc.), rabbit polyclonal anti-eIF2α (FL-315), sc-11386 (Santa Cruz Biotechnology, Inc.), rabbit polyclonal anti-eIF4GI [51] and goat anti-TIA-1 (Acris Antibodies GmbH). Specific antibodies conjugated to Alexa 555 or Alexa 488 were used as secondary antibodies.

## Supporting Information

Figure S1 (4.07 MB EPS)

Figure S2 (8.12 MB EPS)

Figure S3 (6.01 MB EPS)
